# Different low activated clotting time target anticoagulation protocols for patients during extracorporeal membrane oxygenation management: a retrospective cohort study

**DOI:** 10.3389/fmed.2025.1704935

**Published:** 2026-01-12

**Authors:** Fengnian Gu, Ming Zhang, Zhuo Zhang, Junbo Zheng, Liu Jia

**Affiliations:** 1 Harbin Medical University, Harbin, Heilongjiang, China; 2 Department of Critical Care Medicine, the Second Affiliated Hospital of Harbin Medical University, Harbin, Heilongjiang, China

**Keywords:** bleeding, extracorporeal membrane oxygenation, low activated clotting time, oxygenator change, thromboembolism

## Abstract

**Introduction:**

Anticoagulation management is crucial for patients to prevent serious complications during extracorporeal membrane oxygenation (ECMO). However, the optimal target for low activated clotting time (ACT) anticoagulation during ECMO remains unclear.

**Methods:**

A retrospective cohort study was conducted, including patients who received ECMO support in the Second Affiliated Hospital of Harbin Medical University in China from April 2017 to May 2024. Eligible patients were categorized into low (<160 s), medium (160–180 s), and high (>180 s) ACT groups. Major outcomes included ECMO oxygenator change, bleeding and thromboembolic events.

**Results:**

A total of 148 patients were included after applying the exclusion criteria and divided into the low ACT group (*n* = 25, 16.9%), the medium ACT group (*n* = 86, 58.1%), and the high ACT group (*n* = 37, 25.0%). The baseline characteristics were not significantly different among the three groups. The oxygenator change rate did not show a statistically significant difference (low ACT group: 0.0%; medium ACT group: 8.1%; high ACT group: 8.1%; *χ*^2^ = 1.96, *p* = 0.39). There was no significant difference in the proportion of bleeding events among the low (*n* = 3, 12.0%), medium (*n* = 15, 17.4%), and high (*n* = 9, 24.3%) ACT groups (*χ*^2^ = 1.61, *p* = 0.45). The incidence of thromboembolic events showed no significant difference among the low (*n* = 4, 16.0%), medium (*n* = 16, 18.6%), and high (*n* = 9, 24.3%) ACT groups (*χ*^2^ = 0.78, *p* = 0.68).

**Conclusion:**

Different low ACT target anticoagulation protocols for patients during ECMO do not increase the risk of oxygenator change, bleeding and thromboembolism during ECMO management. These results can help clinicians choose appropriate ACT target anticoagulation for patients. Further prospective trials are needed to verify the low ACT target anticoagulation protocols.

**Clinical trial registration:**

Chinese Clinical Trial Registry, ChiCTR2500100151, Registered 3 April, 2025.

## Graphical abstract

**Figure:**
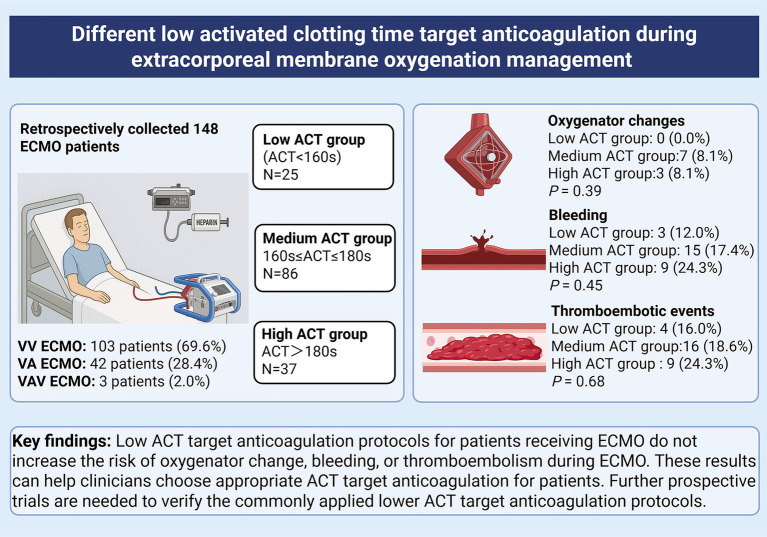

## 1 Introduction

Extracorporeal membrane oxygenation (ECMO) is a supportive therapy that is increasingly being used for patients with acute respiratory or cardiocirculatory failure refractory to medical therapy (1). Anticoagulation is an integral part to maintain the circuit patency, to reduce the risk of thrombosis in the circuit and the patient during ECMO support (2). Unfractionated heparin (UFH) is currently the most commonly used anticoagulant for pediatric and adult ECMO (3). However, heparin usage and the unique clinical situations of individual patients undergoing ECMO treatment may result in many challenges, such as thrombocytopenia or bleeding (4). Therefore, it is widely acknowledged that optimal target anticoagulation management is crucial for patients to prevent serious complications during ECMO.

Although the Extracorporeal Life Support Organization (ELSO) published guidelines in 2017 recommending an activated clotting time (ACT) target at a specified level (typically 1.5 times the normal value for the ACT measurement system) (5), subsequent guidelines in 2021 have highlighted limitations in ACT’s role in ECMO anticoagulation (3). Nevertheless, ACT remains the most frequently used method in many ECMO centers worldwide—largely due to its advantages of rapid bedside results, low cost, and minimal equipment requirements (6). Notably, in clinical practice, different ECMO centers have adopted diverse ACT-based anticoagulation protocols (7). In recent years, a few studies have been conducted to examine the safety and feasibility of low-dose anticoagulation strategies on ECMO patients (8, 9). In a retrospective study of 43 patients, the effect of a lower target ACT (<150 s) during ECMO on safety and outcomes was investigated, compared with those of a conventional target ACT (180–200 s). Their results suggest that a lower target ACT does not necessarily increase the thromboembolic risk during ECMO management (10). Another study including 71 patients with VV ECMO found that a heparin protocol with a lower ACT target of 140–160 s could be feasible compared with the conventional heparin protocol with an ACT target of 180–220 s, and consistently found fewer bleeding events and similar rates of oxygenator changes (11). However, these conclusions require further studies for verification, not only because of the small patient numbers (10, 11), but also because of the use of different low-anticoagulation definitions in different studies (12, 13).

Since in our ECMO center, anticoagulation monitoring methods such as activated partial thromboplastin time (APTT) cannot be performed at the bedside, ACT monitoring remains the primary method for bedside monitoring of UFH therapy. Drawing on our experience in ACT-based anticoagulation management, we have retrospectively reviewed relevant clinical data on ACT-guided anticoagulation for ECMO at our center, and we believe these findings may provide valuable references for clinical ECMO anticoagulation management.

This is the first and largest study to comprehensively evaluate the effect of different low ACT target anticoagulation protocols during ECMO management. We retrospectively compared oxygenator change, bleeding, and thromboembolic events among different ACT target groups.

## 2 Materials and methods

### 2.1 Study design

The trial was conducted in four departments of critical care medicine in the Second Affiliated Hospital of Harbin Medical University in China with an annual ECMO volume of greater than 70 patients. It was a single-center, retrospective cohort study including patients who underwent veno-arterial/veno-venous ECMO(VA/VV ECMO) support between 2017 April to 2024 May. The study design was approved by the medical ethics committee of the Second Affiliated Hospital of Harbin Medical University. The study was registered in the Chinese Clinical Trial Registry as ChiCTR2500100151. Informed consent was not obtained because this retrospective study collected the anonymized electronic medical record data and did not modify diagnostic or therapeutic strategies.

### 2.2 Patients and groups

All patients aged ≥18 years who received ECMO therapy during the study period were included. Exclusion criteria were as follows: (1) ECMO support <24 h, (2) patients who were on ECMO with a no-anticoagulant strategy due to anticoagulation contraindications, such as bleeding. (3) patients who were transferred on ECMO from another hospital, (4) addition of a third cannula within 24 h after ECMO, and (5) pregnancy. Patients were divided into three groups based on the median ACT observed from ECMO day 2 through the entire period until heparin anticoagulation was discontinued. The low ACT group comprised patients with an ACT value less than 160 s. The medium ACT group consisted of patients having ACT values ranging from 160 s to 180 s. The high ACT group included patients with an ACT value greater than 180 s.

### 2.3 ECMO management

The ECMO team in our center assesses the patient’s indication to ascertain the necessity of ECMO support based on the ELSO Guidelines (14, 15). The ECMO systems used were Maquet RotaFlow or CardioHelp with cannulation of the femoral artery and veins or internal jugular veins depending on the ECMO mode, via 17–21 French cannulas.

A bolus of heparin (typically 50–100 units per kilogram) was given just before cannula placement. A target ACT value during ECMO treatment was set by the responsible physician in accordance with the patient’s condition. The infusion rate of heparin was adjusted according to the patient’s target ACT value. ACT was measured every 2–6 h at the bedside (using the Helena Laboratories ACT monitoring device, with a reference range of 120–140 s). In addition to ACT, a range of other laboratory measures were monitored on a daily basis, including international normalized ratio (INR), APTT, fibrinogen, D-dimer, hemoglobin, platelet count, creatinine, and so forth.

The ECMO flows were maintained at a rate that allows for the normal oxygenation and carbon dioxide levels in the blood while minimising the occurrence of hemolysis. Typical flow rates were 3.5 to 4.5 L/min.

The principle of ECMO oxygenator changes in our center includes the following two main aspects. First, an oxygenator change was considered promptly if thrombus was visible to the naked eye in the oxygenator and was affecting the patient’s oxygenation with post-oxygenator PaO₂ < 200 mmHg at FiO₂ 100%. To facilitate the early detection of thrombus, clinicians perform daily rounds of the ECMO circuit. Second, replacement of the circuit could also be considered when repeated clots occur in the oxygenator, resulting in elevated D-dimers with progressive thrombocytopenia(i.e., < 50–80 × 10^9^/L) and hyperfibrinolysis (evidenced by fibrinogen < 2 g/L) (16). Before making a formal decision to change the oxygenator, the ECMO team will carry out a thorough evaluation of the patient and make a final judgement based on the results of the evaluation.

In the transfusion of blood products, a restrictive transfusion strategy was employed, based on a threshold of 7 g/dL for hemoglobin concentration and a platelet count of less than 50,000/mm^3^ (17).

Once the patient’s condition has reached a satisfactory level of improvement and the subsequent criteria have been met, the weaning trial can be initiated following a comprehensive assessment by the physicians according to the ELSO Guidelines (14, 15).

### 2.4 Data collection and outcome measures

Most data were prospectively collected for the ECMO registry in China. Baseline patient characteristics were recorded within the first 24 h after ICU admission, including patients’ demographics, medical history, and laboratory values. After the cannulation of ECMO, relevant clinical information about the ECMO will be recorded, including indications, circuit details, and complications. Other data including ACT, the patient’s other laboratory values, blood products transfusions, and the use of vasoactive drugs, were obtained from the hospital’s electronic database.

Major outcomes were ECMO oxygenator change during the ECMO treatment, bleeding events and thromboembolic events. Bleeding events were defined as newly occurring symptoms of bleeding in any body location during ECMO. All patients underwent routine screening for systemic vascular thrombosis prior to ECMO. Thromboembolic events were defined as newly radiologically confirmed thromboses from ECMO cannulation to 24 h after decannulation, including deep vein thrombosis (DVT), arterial thrombus and pulmonary embolism (PE), identified via ultrasound or other imaging modalities when clinically indicated.

Secondary outcomes included successful weaning rate, ICU mortality rate, hospital mortality rate, ICU length of stay, hospital length of stay, mechanical ventilation time, transfusion requirements, the use of vasoactive medication, the rate of acute kidney injury (AKI), and the need for continuous renal replacement therapy (CRRT) following ECMO support.

### 2.5 Sample size

The sensitivity power analysis was conducted to determine study power (G*Power 3.1.9.4). For the three groups comparison (one-way ANOVA), power calculations (*α* = 0.05, power = 0.80) indicated the sample could detect an effect size of *f* = 0.23. According to Cohen’s standard (1988), *f* = 0.23 is close to a medium effect size (0.10 is small, 0.25 is medium, 0.40 is large).

### 2.6 Statistical analysis

Statistical analysis was performed using SAS software (release 9.13, Serial 989,155; SAS Institute Inc., Shanghai, China). Quantitative variables are reported as mean with standard deviation or median with 25th and 75th percentiles and assessed for normal distribution by the Shapiro–Wilk test. For normally distributed variables, three groups were compared using a one-way analysis of variance (ANOVA). For non-normally distributed variables, the Kruskal-Wallis test was applied, followed by pairwise Wilcoxon rank-sum tests. Qualitative data are described as values or percentages. Groups were compared using Pearson’s chi-square test or Fisher’s exact test based on expected frequencies. A *p*-value < 0.05 was considered to be statistically significant.

## 3 Results

### 3.1 Baseline patient characteristics

**Figure 1:**
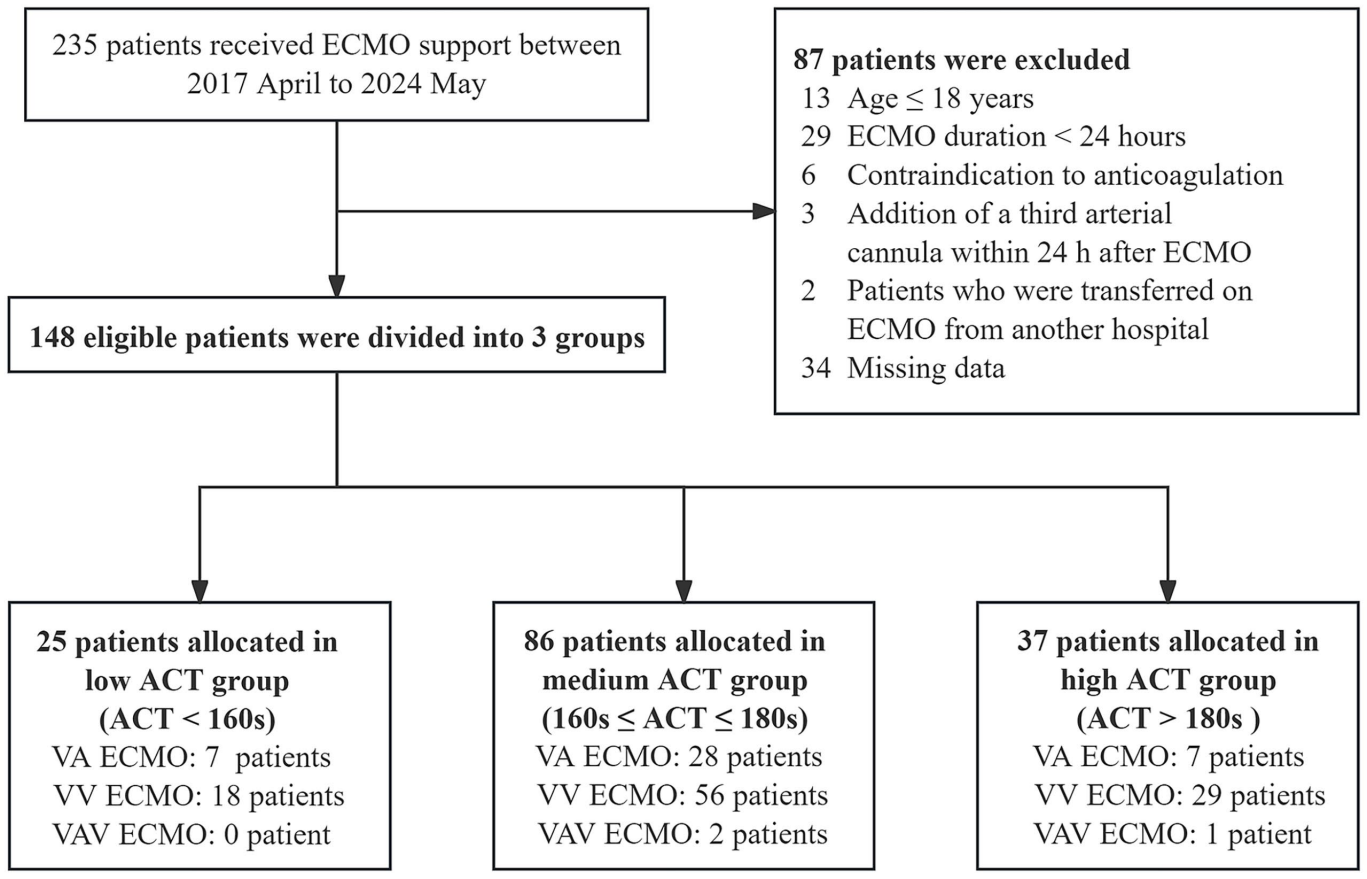
Flow chart for patient cohorts by different ACT groups. ACT, Activated clotting time; ECMO, extracorporeal membrane oxygenation; VA, veno-arterial; VV, veno-venous; VAV, venous-artery and venous–venous.

A total of 235 patients underwent ECMO therapy from April 2017 to May 2024. Patients who met one or more exclusion criteria were excluded (Figure 1). In total, 148 patients were divided into three cohorts: 25 patients in the low ACT group (ACT < 160 s), 86 patients in the medium ACT group (ACT between 160 s and 180 s), and 37 patients in the high ACT group (ACT > 180 s). The baseline characteristics were presented in Table 1, and there were no significant differences among the three groups. The most common comorbidities were hypertension (35.1%), followed by diabetes mellitus (20.3%) and cerebrovascular disease (9.5%). The baseline ACT did not show significant differences among the three groups, *p* = 0.13.

**Table 1:** Baseline patient characteristics.

Patient characteristics	Low ACT group (*n* = 25)	Medium ACT group (*n* = 86)	High ACT group (*n* = 37)	*F/H/χ*^2^	*p*-value
Age, years	59.00 [45.00, 67.00]	57.00 [43.00, 65.00]	57.00 [47.00, 68.00]	2.32	0.31
Gender, *n* (%), male	19(76.0%)	58(67.4%)	27(73.0%)	0.85	0.65
Body weight, kg	70.00 [65.00, 75.00]	71.00 [63.50, 77.75]	68.00 [61.00, 76.00]	0.75	0.69
Height, cm	170.00 [160.00, 176.00]	172.00 [165.00, 176.00]	170.00 [165.00, 173.00]	1.52	0.47
Comorbidities, *n* (%)					
Hypertension	9(36.0%)	28(32.6%)	15(40.5%)	0.73	0.69
Coronary artery disease	3(12.0%)	4(4.7%)	3(8.1%)	2.15	0.31
Diabetes mellitus	8(32.0%)	14(16.3%)	8(21.6%)	3.02	0.22
Cerebrovascular disease	3(12.0%)	6(7.0%)	5(13.5%)	1.83	0.42
Malignancy	1(4.0%)	5(5.8%)	1(2.7%)	0.47	0.87
Aspirin/Warfarin	1(4.0%)	2(2.3%)	2(2.7%)	1.29	0.56
Baseline ACT, seconds	125.00 [114.00, 137.00]	132.00 [123.00, 140.00]	128.00 [121.00, 135.00]	4.02	0.13
Baseline APTT, seconds	32.40 [30.50, 37.30]	32.90 [29.45, 38.88]	35.90 [30.60, 37.60]	1.08	0.58
Baseline PT-INR	1.10 [1.04, 1.24]	1.15 [1.05, 1.28]	1.18 [1.07, 1.31]	1.88	0.39
Baseline fibrinogen, g/L	3.54 [2.73, 4.70]	3.69 [2.75, 4.85]	3.74 [2.63, 5.00]	0.61	0.74
Baseline d-dimers, mg/L	1174.00 [390.00, 2777.00]	993.50 [382.75, 3235.75]	1151.00 [538.00, 2737.00]	0.04	0.98
Baseline PLT, thousand/μL	201.00 [154.00, 255.00]	197.00 [123.25, 249.50]	178.00 [122.00, 234.00]	0.70	0.71
Baseline WBC, thousand/μL	9.70 [6.90, 16.10]	10.20 [6.12, 15.80]	7.60 [5.10, 13.10]	2.28	0.32
Baseline Hemoglobin, mg/dL	125.00 [113.00, 142.00]	130.00 [110.25, 146.75]	126.00 [111.00, 138.00]	0.95	0.62
Baseline TB, mg/dL	12.90 [11.00, 19.50]	18.20 [10.75, 24.95]	15.50 [9.10, 28.00]	1.08	0.58
Baseline Creatinine, mg/dL	93.00 [78.00, 179.00]	90.50 [71.25, 123.75]	96.00 [64.00, 145.00]	2.14	0.34

ACT, Activated clotting time; APTT, Activated partial thromboplastin time; PT-INR, Prothrombin time-international normalized ratio; PLT, Thrombocyte count; WBC, White blood cells; TB, Total bilirubin. ^*^p < 0.05.

### 3.2 ECMO related characteristics

ECMO related characteristics were evaluated (Table 2). There was no statistical difference in indications for ECMO among groups (*p* = 0.32). It should be noted that the most common indication was respiratory disease (73.6%), including pneumonia (67.6%), COVID-19 (4.7%), and pulmonary fibrosis (1.4%). This was followed by circulatory diseases (22.2%) included aortic dissection (3.4%), myocardial infarction (2.7%) and myocarditis (2.7%). Other indications (4.1%) were digestive system infections (3.4%) and ketoacidosis (0.7%). Of the 148 patients who underwent ECMO therapy, 42 were treated with veno-arterial ECMO, 103 with venous–venous ECMO, and 3 with venous-artery-venous ECMO. The distribution of ECMO revolutions per minute (rpm) and flow was homogeneous among the groups (*p* = 0.37, *p* = 0.87, respectively). Since the initial preset of grouping, the median ACT value was the lowest in low ACT group (155.00 [152.00, 158.00]) followed by medium ACT group (170.00 [166.00, 175.38]) and high ACT group (185.00 [182.00, 193.00]), *p* < 0.01. The maximum ACT on ECMO day 1 did not differ significantly across groups (low: 195.00 [178.00, 236.00], medium: 209.00 [190.00, 236.00], high: 205.00 [190.00, 249.00]; *p* = 0.30). The median APTT values were statistically significant among three groups: low ACT group (43.50 [40.80, 54.85]), medium ACT group (50.75 [42.79, 55.86]) and high ACT group (58.70 [49.20, 62.45]), (*p* < 0.01).

**Table 2:** ECMO related and laboratory variables.

Variables	Low ACT group (*n* = 25)	Medium ACT group (*n* = 86)	High ACT group (*n* = 37)	*F*/*H*/*χ*^2^	*p*-value
ECMO indication				4.41	0.32
Respiratory	18 (72.0%)	60 (69.8%)	31 (83.8%)		
Circulatory	6 (24.0%)	23 (26.7%)	4 (10.8%)		
Others	1 (4.0%)	3 (3.5%)	2 (5.4%)		
ECMO mode				2.94	0.52
Venous-artery	7 (28.0%)	28 (32.6%)	7 (18.9%)		
Venous–venous	18 (72.0%)	56 (65.1%)	29 (78.3%)		
Venous-artery-venous	0 (0.0%)	2 (2.3%)	1 (2.7%)		
ECMO rpm	3521.80 ± 357.84	3532.38 ± 421.33	3422.84 ± 376.12	1.00	0.37
ECMO flow, liter per minute	3.70 [3.50, 4.00]	3.65 [3.42, 4.00]	3.80 [3.50, 4.00]	0.27	0.87
ECMO runtime, hours	190.00 [144.00, 292.00]	173.50 [116.25, 269.25]	171.00 [121.00, 281.00]	1.37	0.51
Median ACT, seconds	155.00 [152.00, 158.00]	170.00 [166.00, 175.38]	185.00 [182.00, 193.00]	115.23	<0.01^*^
Maximum ACT on ECMO day 1	195.00 [178.00, 236.00]	209.00 [190.00, 236.00]	205.00 [190.00, 249.00]	2.42	0.30
Median APTT, seconds	43.50 [40.80, 54.85]	50.75 [42.79, 55.86]	58.70 [49.20, 62.45]	15.39	<0.01^*^
Median PT-INR	1.21 [1.08, 1.35]	1.26 [1.13, 1.46]	1.24 [1.14, 1.35]	2.29	0.32
Median fibrinogen, g/L	2.73 ± 0.99	3.00 ± 1.04	2.89 ± 1.13	0.45	0.64
Median PLT, thousand/μL	102.00 [75.00, 123.00]	98.00 [57.00, 144.00]	97.00 [59.50, 133.38]	0.23	0.89
Median WBC, thousand/μL	8.80 [6.45, 12.50]	9.80 [7.70, 12.50]	9.35 [7.00, 12.65]	0.79	0.67
Median Hemoglobin, mg/dL	80.00 [75.50, 92.00]	81.50 [72.00, 96.50]	82.00 [75.00, 91.00]	0.02	0.99
Median TB, mg/dL	18.20 [14.15, 42.30]	21.45 [13.80, 30.20]	24.20 [19.04, 40.52]	3.40	0.18
Median Creatinine, μmol/L	97.00 [79.00, 163.00]	106.00 [77.50, 183.00]	97.75 [65.12, 146.00]	1.59	0.45

ACT, Activated clotting time; ECMO, Extracorporeal membrane oxygenation; RPM, Revolutions per minute; APTT, Activated partial thromboplastin time; PT-INR, Prothrombin time-international normalized ratio; PLT, Thrombocyte count; WBC, White blood cells; TB, Total bilirubin. ^*^p < 0.05.

### 3.3 Outcomes

**Figure 2:**
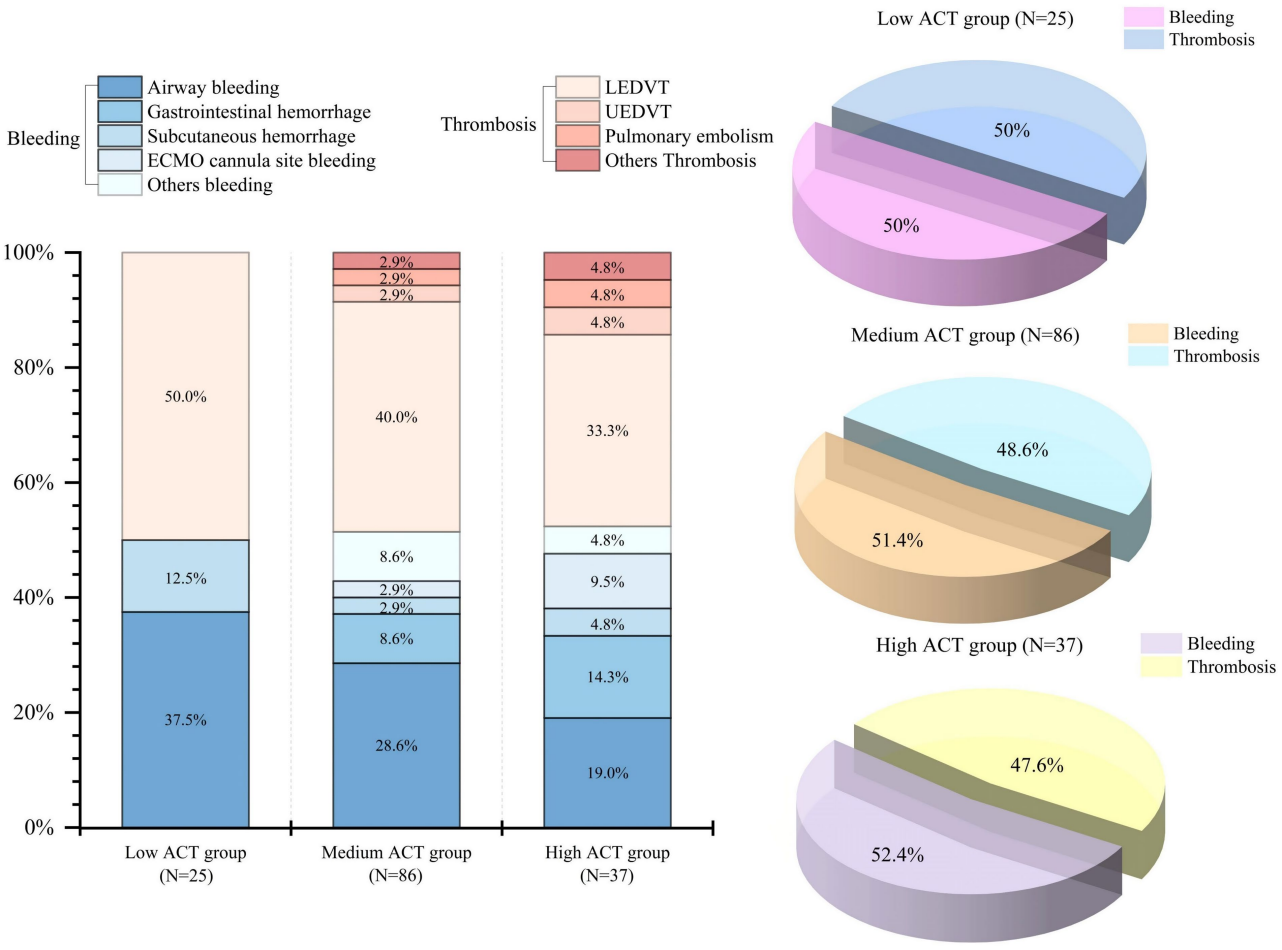
Distribution of bleeding and thrombotic events across different ACT groups. ACT, Activated clotting time; LEDVT, Lower extremity deep vein thrombosis; UEDVT, Upper extremity deep vein thrombosis.

Table 3 and Figure 2 show the major and secondary outcomes of the patients.

**Table 3:** Major and secondary outcomes.

Variables	Low ACT group (*n* = 25)	Medium ACT group (*n* = 86)	High ACT group (*n* = 37)	*F/H/χ*^2^	*p*-value
Major outcomes					
Oxygenator change	0 (0.0%)	7 (8.1%)	3 (8.1%)	1.96	0.39
Bleeding events	3 (12.0%)	15 (17.4%)	9 (24.3%)	1.61	0.45
Airway	3 (12.0%)	10 (11.6%)	4 (10.8%)	0.13	0.99
Gastrointestinal hemorrhage	0 (0.0%)	3 (3.5%)	3 (8.1%)	2.15	0.36
Subcutaneous hemorrhage	1 (4.0%)	1 (1.2%)	1 (2.7%)	1.67	0.38
ECMO cannula site bleeding	0 (0.0%)	1 (1.2%)	2 (5.4%)	2.32	0.23
Others	0 (0.0%)	3 (3.5%)	1 (2.7%)	0.94	0.81
Thromboembolic events	4 (16.0%)	16 (18.6%)	9 (24.3%)	0.78	0.68
LEDVT	4 (16.0%)	14 (16.3%)	7 (18.9%)	0.15	0.93
UEDVT	0 (0.0%)	1 (1.2%)	1 (2.7%)	1.13	0.66
Pulmonary embolism	0 (0.0%)	1 (1.2%)	1 (2.7%)	1.13	0.66
Others	0 (0.0%)	1 (1.2%)	1 (2.7%)	1.13	0.66
Secondary outcomes					
Success weaning off	10 (40.0%)	49 (57.0%)	20 (54.1%)	3.25	0.32
ICU mortality	9 (36.0%)	41 (47.7%)	15 (40.5%)	1.30	0.52
Hospital mortality	16 (64.0%)	51 (59.3%)	18 (48.6%)	1.73	0.42
ICU LOS, days	13.00 [8.00, 23.00]	15.00 [9.00, 24.75]	14.00 [11.00, 23.00]	0.32	0.85
Hospital LOS, days	19.00 [16.00, 26.00]	23.50 [12.00, 33.50]	19.00 [13.00, 29.00]	0.36	0.84
MV time, hours	197.00 [123.00, 414.00]	261.50 [138.25, 409.50]	235.00 [160.00, 359.00]	0.07	0.97
CRRT	10 (40.0%)	40 (46.5%)	18 (48.7%)	0.48	0.79
AKI during ECMO	0 (0.0%)	4 (4.7%)	1(2.7%)	0.78	0.83
Use of vasoactive medication					
Epinephrine	14 (56.0%)	46 (53.5%)	27(73.0%)	4.15	0.13
Norepinephrine	13 (52.0%)	46 (53.5%)	24(64.9%)	1.56	0.46
Milrinone	9 (36.0%)	24 (27.9%)	9(24.3%)	1.02	0.60
Dopamine	11 (44.0%)	31 (36.1%)	18(48.7%)	1.85	0.40
Dobutamine	7 (28.0%)	16 (18.6%)	7(18.9%)	1.11	0.57
Vasopressin	14 (56.0%)	45 (52.3%)	22(59.4%)	0.55	0.76
Blood products transfusion					
FFP, mL	600.00 [0.00, 1980.00]	200.00 [0.00, 1890.00]	400.00 [0.00, 1600.00]	0.09	0.96
PRBC, units	2.00 [0.00, 8.00]	0.00 [0.00, 6.00]	2.00 [0.00, 12.00]	1.17	0.56
Platelet, units	0.00 [0.00, 0.50]	0.00 [0.00, 1.00]	0.00 [0.00, 1.50]	0.23	0.89

ACT, Activated clotting time; ECMO, Extracorporeal membrane oxygenation; LEDVT, Lower extremity deep vein thrombosis; UEDVT, Upper extremity deep vein thrombosis; ICU, Intensive care unit; LOS, Length of stay; MV, Mechanical ventilation; CRRT, Continuous renal replacement therapy; AKI, Acute kidney injury; FFP, Fresh frozen plasma; PRBC, Packed red blood cell.

#### 3.3.1 Major outcomes

Overall, there was no oxygenator change in the low ACT group. Although the oxygenator change rate was comparatively lower in the low ACT group (0.0%) than in the medium ACT group (8.1%) and the high ACT group (8.1%), no statistically significant difference was observed among the three groups (*χ*^2^ = 1.96, *p* = 0.39) (Table 3).

Bleeding occurred in 27 (18.2%) of patients in the study, and airway bleeding was the most common type (Figure 2). There was a lower proportion of bleeding events observed in the low ACT group (12.0%) when compared to the medium ACT group (17.4%) and the high ACT group (24.3%). However, the difference among these groups was not statistically significant (*p* = 0.45) (Table 3).

The overall incidence of thromboembolic events was 19.6% (29 patients) and lower extremity deep vein thrombosis (LEDVT) was the primary thrombotic complication (Figure 2). The incidence did not show a statistically significant difference among the three groups (*p* = 0.68). Specifically, there were 4 patients (16.0%) in the low ACT group, 16 patients (18.6%) in the medium ACT group, and 9 patients (24.3%) in the high ACT group. Of note, 2 patients were diagnosed with pulmonary embolism: one in the medium ACT group and one in the high ACT group. Additionally, 2 patients developed thrombi at other sites: one in the internal jugular vein (the medium ACT group) and the other at the cardiac apex (the high ACT group).

#### 3.3.2 Secondary outcomes

The successfully weaned off ECMO rate was similar among the three groups (*χ*^2^ = 3.25, *p* = 0.32). Similarly, the ICU and hospital mortality did not show statistically significant differences among the three groups (*χ*^2^ = 1.30, *p* = 0.52; *χ*^2^ = 1.73, *p* = 0.42, respectively). No significant differences were observed in the use of vasoactive drugs and other clinical outcomes, including the ICU and hospital length of stay, mechanical ventilation duration, the transfusion of blood products, the rate of AKI, and the need for CRRT following ECMO support.

## 4 Discussion

Our study is the first and largest to comprehensively evaluate the effects of multiple ACT target anticoagulation protocols during ECMO management, providing novel evidence for clinical practice. Five previous reports on ECMO anticoagulation included more patients than our study (18–22). However, only one study was related to ACT. Seeliger et al. analyzed 218 VV-ECMO patients in a retrospective observational cohort study, comparing low-dose heparinization (target APTT: 35–40 s) with high-dose therapeutic heparinization (target ACT: 140–180 s). They found that high-dose heparinization was associated with lower rates of oxygenator changes and thromboembolic events (18).

Of the 148 patients included in our study, the major outcomes (oxygenator changes, bleeding and thromboembolic events) did not show significant differences among the three groups. Nevertheless, these findings showed that there was no apparent harm from lower anticoagulation targets for ECMO patients. However, it is important to note that ACT measurements can vary between devices and institutions due to differences in testing platforms and protocols. Furthermore, as this is a retrospective study, the clinicians may have picked a low anticoagulation strategy for high bleeding risk patients, and a high anticoagulation strategy for high thrombotic risk patients when ECMO was initiated. Therefore, its clinical utility requires further investigation and local validation.

### 4.1 Low anticoagulation strategies during ECMO support

Recently, an increasing number of researchers have been focusing on low anticoagulation strategies for patients undergoing ECMO support to reduce the risk of bleeding complications (23, 24). However, the main question was that different low-anticoagulation definitions were used in different studies, highlighting a lack of consensus in clinical practice.

First, different monitoring methods have been used in previous studies on low anticoagulation, such as ACT (10, 11), APTT (20, 21), and anti-factor Xa (25, 26). Second, different studies have defined varying thresholds for the same monitoring values for low anticoagulation. Third, different anticoagulation monitoring values, such as ACT and activated partial thromboplastin time (APTT), were compared (12, 18). Based on these findings, the clinical effect of low anticoagulation strategies for patients undergoing ECMO support was not entirely consistent. Moreover, some researchers regarded the strategy without continuous systemic anticoagulation as a form of low anticoagulation, and they found that heparin-free anticoagulation was safe in patients supported by ECMO (13, 27).

Therefore, standardizing the definition of low anticoagulation will facilitate more accurate clinical outcome evaluations in the future. For example, anticoagulation levels below the range recommended by the ELSO guidelines (ACT 180–210 s), defined as low intensity anticoagulation, may be more reasonable. Similarly, APTT and anti-factor Xa activity should also have consistent standard ranges for low anticoagulation.

In our ECMO center, physicians commonly utilize low anticoagulation protocols for patients receiving ECMO. In this study, 111 patients were classified into the low and medium ACT groups, with ACT targets below the guideline-recommended targets. Overall, the oxygenator replacement rate (6.8%) was lower than that in previous studies (16, 28), including the larger one on oxygenator replacement (29), thereby reflecting the diverse practices of oxygenator replacement across different ECMO centers. The bleeding rate and thromboembolic rate in our cohort were lower than those reported in large ECMO registries (23.4 and 25.3%, respectively) (30). Furthermore, there were no significant differences in oxygenator replacement rates, bleeding rates, and thromboembolic rates among the study groups, validating the feasibility of these low anticoagulation strategies. These findings were similarly reported in previous studies (25) and require further prospective research to confirm.

However, to the best of our knowledge, there remains a lack of prospective randomized controlled trials (RCTs) that validate the efficacy and safety of low anticoagulation strategies in patients undergoing ECMO. A pilot RCT compared therapeutic anticoagulation with heparin (target APTT between 50 and 70 s) versus lower dose heparin (target APTT < 45 s) among 32 patients receiving venoarterial or venovenous ECMO in two hospitals. They found that allocating patients on ECMO to two different anticoagulation protocols led to a significant difference in mean daily APTT and anti-Xa levels between groups. The results support the feasibility of a larger trial in patients undergoing ECMO to compare different anticoagulation protocols (31). Recently, a multicenter, randomized pilot trial was conducted at three centers across the United States and compared low-intensity anticoagulation with an intermittent, subcutaneous administration of an anticoagulant at the doses used for deep venous thrombosis prophylaxis versus moderate-intensity anticoagulation with a continuous, goal-titrated, infusion of anticoagulant targeting a goal APTT of 40–60 s or a goal anti-Xa level of 0.2 to 0.3 IU/mL during venovenous ECMO. However, only 26 patients were enrolled and no conclusions regarding efficacy or safety can be made (32). Currently, the RATE trial, a larger RCT in adult patients treated with ECMO, is ongoing (33, 34). They hypothesized that with lower anticoagulation targets or anticoagulation with low molecular weight heparin during ECMO therapy, patients would have fewer hemorrhagic complications without an increase in thromboembolic complications or a negative effect on their outcome. It is worth noting that the low anticoagulation target in their study refers to 1.5–2 times baseline APTT (about 45–60 s). In fact, these RCTs still involve the problem of a lack of a unified low-anticoagulation standard. We look forward to the findings of this research, and more high-quality RCTs are needed in the future.

### 4.2 Personalized anticoagulation strategies during ECMO support

Instead of setting a fixed standardized anticoagulation target for the patients during ECMO support, personalized anticoagulation strategies may be of greater importance. In contrast, mechanical application of uniform anticoagulation targets may lead to over-anticoagulation. Anticoagulation targets determined based on patients’ baseline anticoagulation monitoring values might be more reasonable. Because ACT in particular has the advantage of being inexpensive and convenient and is difficult to be replaced in developing countries (6), we chose it as an example. When a patient’s baseline ACT level is 130 s, setting the ACT target at about 200 s may be appropriate for this particular patient. In our study, the majority of patients exhibited baseline ACT values within the conventional range of 120–140 s. However, 26 patients (17.6%) demonstrated baseline ACT values below 120 s. Furthermore, the lowest baseline ACT value among patients was 90 s, and setting an anticoagulation target of ACT 135 s for this patient may be a suitable choice. If the ACT exceeds 150 s, although it is still within the target range, it may indicate a state of excessive anticoagulation, increasing the risk of bleeding. Otherwise, if a patient’s baseline ACT has already exceeded the normal range, a low anticoagulation strategy should be adopted. Therefore, we propose that anticoagulation targets should be more precisely tailored to each patient’s baseline coagulation status and patient-specific risk factors to optimize the efficacy and minimize associated risks. Further work is needed to evaluate the use of personalized anticoagulation strategies during ECMO support.

### 4.3 Study limitations

The present study has several limitations as a retrospective study. First, bleeding events, especially mild bleeding at the site of ECMO cannulation, may not be fully recorded. Second, not all doctors routinely performed ultrasound examinations after decannulation to assess thrombotic events. Additionally, the clot formation on the ECMO oxygenator was poorly documented. As a result, the incidence of bleeding and thrombotic events may have been underestimated. Third, our study primarily included patients receiving VV-ECMO, whereas VA-ECMO is associated with distinct hemodynamic conditions and higher thrombotic complication rates (35, 36). Consequently, the generalizability of our findings to VA-ECMO patients may be limited. Another limitation is that no systemic anticoagulation should be considered a low-anticoagulation strategy. However, in our center, this approach was only used for patients with contraindications to anticoagulation therapy, and the number of such cases was relatively limited. Therefore, we did not evaluate the use of no systemic anticoagulation in this study. Although we conducted sensitivity power analysis and found that the current sample size had the capacity to detect an effect size approaching a moderate level, the current sample sizes are still small and future validation studies should further expand the sample size.

## 5 Conclusion

Low ACT target anticoagulation protocols for patients receiving ECMO do not increase the risk of oxygenator change, bleeding, or thromboembolism during ECMO management. These results can help clinicians choose appropriate ACT target anticoagulation for patients. Further prospective trials are needed to verify the commonly applied low ACT target anticoagulation protocols.

## Data Availability

The original contributions presented in the study are included in the article/supplementary material, further inquiries can be directed to the corresponding author.
